# P328: Descriptive study and proposal for improving the management of expired medicines in developing countries: the case of Benin

**DOI:** 10.1186/2047-2994-2-S1-P328

**Published:** 2013-06-20

**Authors:** K Stéphanie, Marie De Solere

**Affiliations:** 1 University of Bordeaux 2, Bordeaux, France

## Introduction

In countries with limited resources, access to quality medicines is a major public health challenge. Pharmaciens Sans Frontières Indre et Loire (PSF37) is a French association who has been working on this issue for several years in Benin. During their missions, PSF37 members have noticed a lack in the management of expired drugs, which can cause a real problem of accumulation and a risk of using these drugs.

## Objectives

We chose to study this matter in the region of Borgou-Alibori in Benin, by analyzing the management of expired drugs in the public pharmaceutical chain.

## Methods

A cross-sectional survey in the different levels of the supply chain was conducted through semi-structured interviews, questionnaires and observation checklists. The study covered 31 health facilities of Borgou-Alibori: hospitals, warehouses and 16 health centers. The national and departmental health has also been interviewed.

## Results

The amount of expired drugs found in both pharmacies' common stocks and storage areas show that there is in Benin, a problem related to the accumulation of these products. The lack of rigor in the management of medicines, the lack of qualified staff and the lack of regulations governing professional practices have been identified as major causes of this problem.

## Conclusion

In order to initiate an improvement process we proposed at the end of this study and on behalf of PSF37, an action plan to Benin authorities.

## Disclosure of interest

None declared.

